# Involvement of GLUT1-mediated glucose transport and metabolism in gefitinib resistance of non-small-cell lung cancer cells

**DOI:** 10.18632/oncotarget.25994

**Published:** 2018-08-24

**Authors:** Shuhei Suzuki, Masashi Okada, Hiroyuki Takeda, Kenta Kuramoto, Tomomi Sanomachi, Keita Togashi, Shizuka Seino, Masahiro Yamamoto, Takashi Yoshioka, Chifumi Kitanaka

**Affiliations:** ^1^ Department of Molecular Cancer Science, Yamagata University School of Medicine, Yamagata 990-9585, Japan; ^2^ Department of Clinical Oncology, Yamagata University School of Medicine, Yamagata 990-9585, Japan; ^3^ Department of Ophthalmology, Yamagata University School of Medicine, Yamagata 990-9585, Japan; ^4^ Research Institute for Promotion of Medical Sciences, Yamagata University Faculty of Medicine, Yamagata 990-9585, Japan

**Keywords:** non-small-cell lung cancer (NSCLC), epidermal growth factor receptor (EGFR), tyrosine kinase inhibitor (TKI), glycolysis, animal model

## Abstract

Use of epidermal growth factor receptor (EGFR) inhibitors represented by gefitinib and erlotinib has become the standard of treatment for non-small-cell lung cancers (NSCLCs) with activating EGFR mutations. However, the majority of NSCLCs, which overexpress EGFR without such mutations, are resistant to EGFR inhibitors, and the mechanism(s) behind such primary resistance of NSCLCs without activating EGFR mutations to EGFR inhibitors still remains poorly understood. Here in this study, we show that glucose metabolism mediated by GLUT1, a facilitative glucose transporter, is involved in gefitinib resistance of NSCLC cells. We found that GLUT1 expression and glucose uptake were increased in resistant NSCLC cells after gefitinib treatment and that genetic as well as pharmacological inhibition of GLUT1 sensitized not only NSCLC cells with primary resistance but also those with acquired resistance to gefitinib. *In vivo*, the combination of systemic gefitinib and a GLUT1 inhibitor, both of which failed to inhibit tumor growth when administered alone, significantly inhibited the growth of xenograft tumors formed by the implantation of NSCLC cells with wild-type EGFR (wt-EGFR). Since our data indicated that GLUT1 was similarly involved in erlotinib resistance, our findings suggest that the activity of GLUT1-mediated glucose metabolism could be a critical determinant for the sensitivity of NSCLC cells to EGFR inhibitors and that concurrent GLUT1 inhibition may therefore be a mechanism-based approach to treating NSCLCs resistant to EGFR inhibitors, including those with wt-EGFR.

## INTRODUCTION

Non-small cell lung cancer (NSCLC) accounts for approximately 80% of lung cancer, the leading cause of cancer death worldwide [1, 2]. Since the identification of specific epidermal growth factor receptor (EGFR) mutations as a critical predictor of therapeutic response of NSCLC patients to gefitinib, an ATP-competitive EGFR tyrosine kinase inhibitor (EGFR-TKI), gefitinib and other recently developed EGFR inhibitors have been the mainstay in the management of NSCLC with activating EGFR mutations [3, 4]. However, in contrast to the dramatic improvement achieved by EGFR inhibitors in the survival of patients with NSCLC harboring activating EGFR mutations, EGFR inhibitors still have only limited roles in the clinical management of patients with NSCLC lacking such mutations, which constitutes the majority of NSCLC cases [5, 6, 7]. Understanding the mechanisms behind the resistance of NSCLCs without activating EGFR mutations to EGFR inhibitors is therefore considered crucial to further improve the efficacy of EGFR inhibitors in NSCLC, but the details as to how the absence of such mutations render NSCLC resistant to EGFR inhibitors still remain largely unknown [8, 9, 10].

Here in this study, we investigated the role of glucose metabolism as a possible determinant for the sensitivity of NSCLC to gefitinib. Our results suggest that glucose metabolism mediated by a facilitative glucose transporter GLUT1 may be critically involved in the gefitinib resistance of NSCLC cells without activating mutations and that GLUT1 inhibition may become a rational approach to sensitizing otherwise resistant NSCLC to gefitinib and other EGFR inhibitors.

## RESULTS

### Increased glucose uptake associated with GLUT1 expression after gefitinib treatment in EGFR-wt NSCLC cells and in EGFR-mutated NSCLC cells with acquired gefitinib resistance

**Figure 1:**
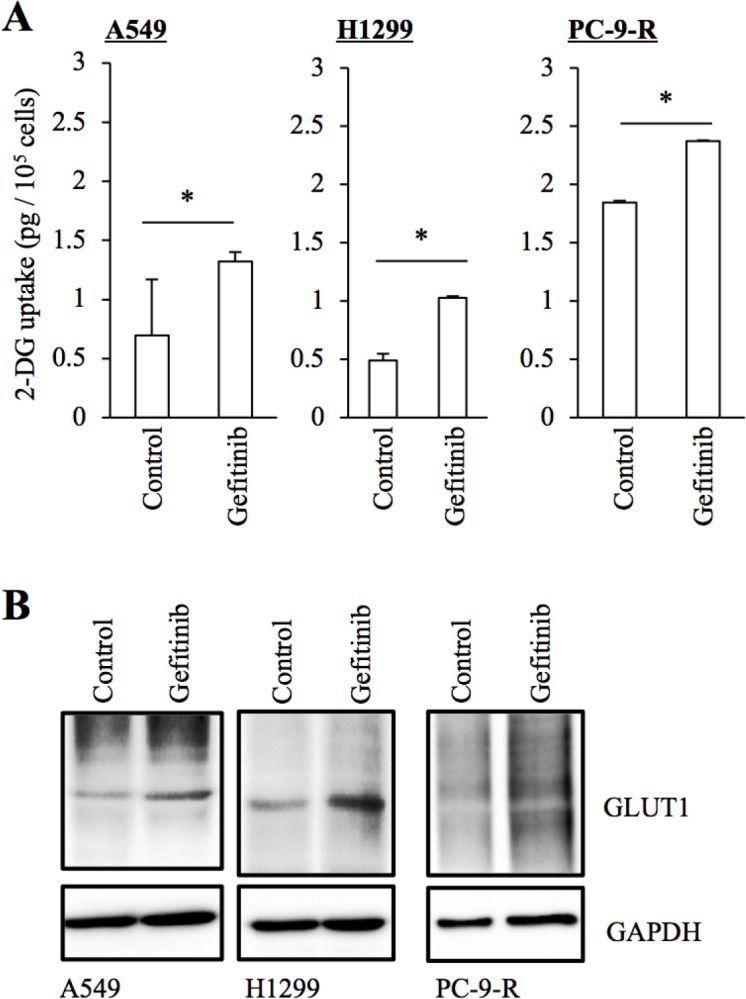
Increased GLUT1 expression and glucose uptake in NSCLC cells treated with gefitinib The indicated non-small-cell lung cancer (NSCLC) cells cultured in the absence (Control) and presence of 10 μM gefitinib for 3 days were subjected to glucose uptake assay **(A)** and immunoblot analysis of GLUT1 expression **(B)**. Values in the graphs represent means + SD from three independent experiments. ^*^P < 0.05.

As an initial approach to understanding the mechanisms involved in the differential sensitivity of EGFR-wt and EGFR-mutated NSCLC cells to EGFR-TKIs such as gefitinib and erlotinib, we focused on glucose metabolism, since previous studies suggested that glucose metabolism regulated by EGFR may mediate the therapeutic effects of EGFR-TKIs in EGFR-mutated NSCLC cells [11, 12]. First, we tested the effect of gefitinib on glucose uptake by A549 and H1299, NSCLC cell lines that express wt-EGFR and are resistant to gefitinib. It has been shown earlier that erlotinib inhibits glucose uptake by mutant EGFR-expressing NSCLC cells sensitive to EGFR-TKIs [11]. However, we found that glucose uptake was rather increased in A549 and H1299 cells treated with gefitinib. Significantly, PC-9-R, a gefitinib-resistant subline derived from PC-9 (a mutant EGFR-expressing NSCLC cell line sensitive to EGFR-TKIs), showed a higher basal level of glucose uptake compared to that of PC-9 (1.8 pg/10^5^ cells for PC-9-R and 0.56 pg/10^5^ cells for PC-9), which was further increased after gefitinib treatment (Figure 1A). Given that GLUT1, a facilitative glucose transporter, reportedly mediates glucose metabolism in EGFR-mutated NSCLC cells [12], we also determined the expression levels of GLUT1 in these NSCLC cells with and without gefitinib treatment. We found that, in association with their glucose uptake activities, the expression levels of GLUT1 were increased in cells treated with gefitinib (Figure 1B). Together, these results suggested the possibility that gefitinib resistance may be associated with concomitant increase in GLUT1 expression and glucose uptake.

### Pharmacological inhibition of GLUT1 sensitizes resistant NSCLC cells to gefitinib

**Figure 2:**
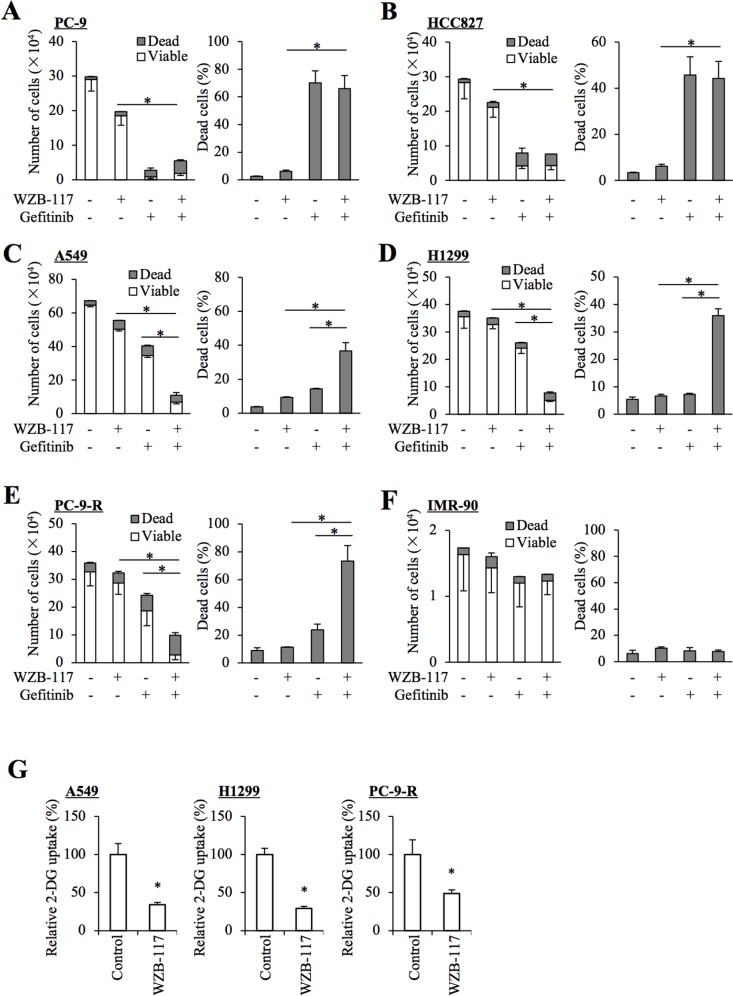
Pharmacological inhibition of GLUT1 by WZB-117 sensitizes resistant NSCLC cells to gefitinib at a concentration non-toxic to normal cells The indicated non-small-cell lung cancer (NSCLC) cells **(A–E)**, 1 × 10^5^ and IMR-90 normal human fibroblasts **(F)**, 1 × 10^4^ were treated with or without 10 μM gefitinib in the presence or absence of 7.5 μM WZB-117 for 3 days and then subjected to cell viability assay to determine the numbers of viable and dead cells (left panels) as well as the percentage of dead cells (right panels). **(G)** The indicated NSCLC cells treated with or without 7.5 μM WZB-117 for 2 h were subjected to glucose uptake assay. Values in the graphs represent means and SD from three independent experiments. ^*^P < 0.05 [note that, in the left panels of A through F, it is the numbers of viable cells that are compared].

Prompted by the observation that GLUT1 expression and glucose uptake are increased in gefitinib-resistant NSCLC cells, we next examined the impact of GLUT1 inhibition on the sensitivity/resistance of NSCLC cells to gefitinib by use of WZB-117, a pharmacological inhibitor of GLUT1 [13, 14]. Consistent with earlier reports [15, 16, 17], treatment with 10 μM gefitinib, which efficiently inhibited the growth of NSCLC cells with activating mutations (PC-9 and HCC827, Figure 2A and 2B), only modestly or marginally inhibited the growth in NSCLC cells with wt-EGFR (A549 and H1299, Figure 2C and 2D). However, in the presence of WZB-117 at a concentration (7.5 μM) sufficient to reduce glucose uptake in NSCLC cells ([13], and Figure 2G), gefitinib inhibited cell growth significantly more efficiently in these cells accompanied by an apparent increase in the proportion of dead cells (Figure 2C and 2D). Importantly, the combinatorial treatment with gefitinib and WZB-117 inhibited the growth of PC-9-R cells far more efficiently than either alone (Figure 2E), whereas the same combination (and either treatment alone) showed no growth-inhibitory effect on IMR-90 human fetal lung fibroblasts (Figure 2F). These results suggested that glucose metabolism mediated by intracellular glucose transport through GLUT1 may be involved in gefitinib resistance of NSCLC cells and that the combination of gefitinib and GLUT1 inhibition may have a selective growth-inhibitory effect on NSCLC cells.

### Genetic knockdown of GLUT1 sensitizes resistant NSCLC cells to gefitinib

**Figure 3:**
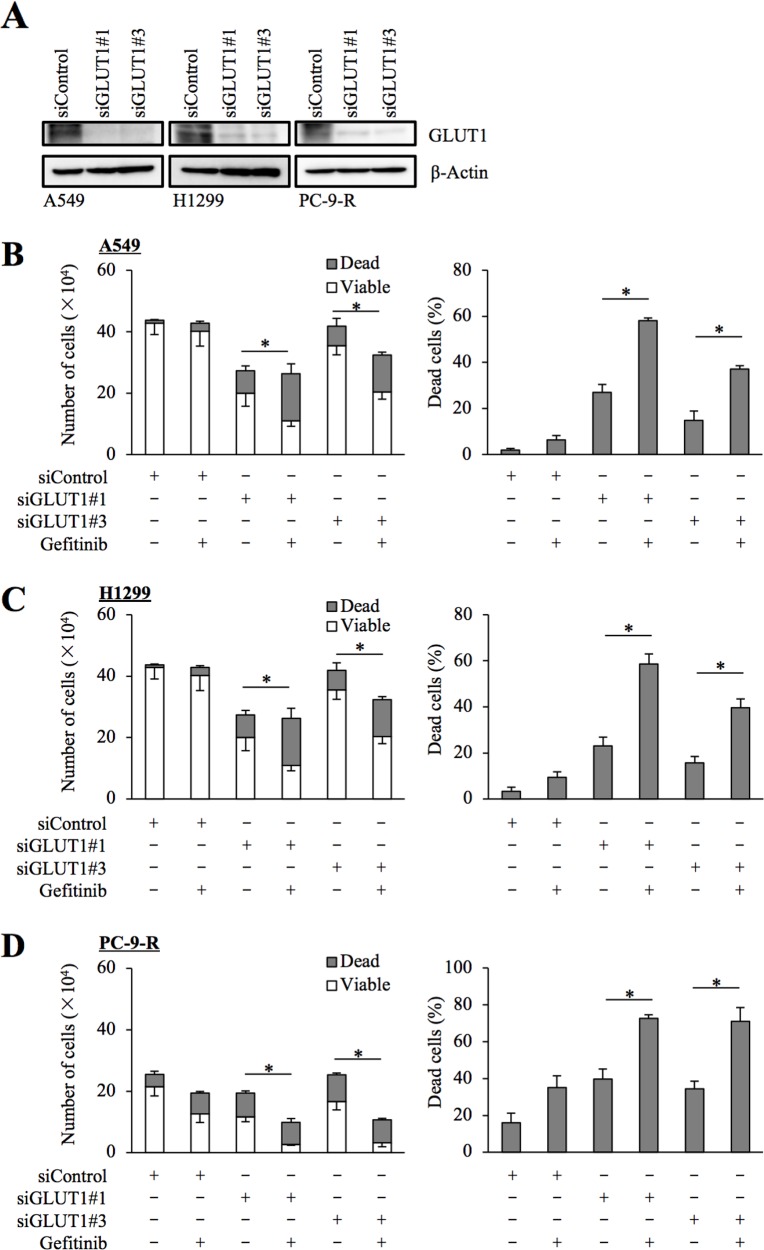
siRNA-mediated knockdown of GLUT1 sensitizes resistant NSCLC cells to gefitinib The indicated non-small-cell lung cancer (NSCLC) cells were transfected with a non-targeting siRNA (siControl) or either of the siRNAs against GLUT1 (siGLUT1#1 and siGLUT1#3) for 3 days. The cells were then subjected to immunoblot analysis of GLUT1 protein expression **(A)**, or alternatively, treated with 10 μM gefitinib for another 3 days and subjected to cell viability assay to determine the numbers of viable and dead cells (left panels) as well as the percentage of dead cells (right panels) **(B–D)**. Values in the graphs represent means and SD from three independent experiments. ^*^P < 0.05 [note that the numbers of viable cells are compared in the left panels of B through D].

To exclude the possibility that WZB-117 sensitized NSCLC cells to gefitinib through an off-target mechanism, we next conducted GLUT1 knockdown experiments. Introduction of either of two different siRNAs against GLUT1, but not a non-targeting siRNA, resulted in decreased GLUT1 expression in NSCLC cells (Figure 3A). Under this experimental condition, knockdown of GLUT1 in gefitinib-resistant NSCLC cells by either siRNA caused, similarly to WZB-117 treatment, a modest inhibition of cell growth compared to control knockdown. Gefitinib treatment further decreased the number of viable cells and increased the proportion of dead cells in GLUT1 knockdown cells but not in control cells, indicating that GLUT1 expression is indeed required for the gefitinib resistance of gefitinib-resistant cells (Figure 3B–3D).

### Inhibition of the initial step of glycolysis sensitizes resistant NSCLC cells to gefitinib

**Figure 4:**
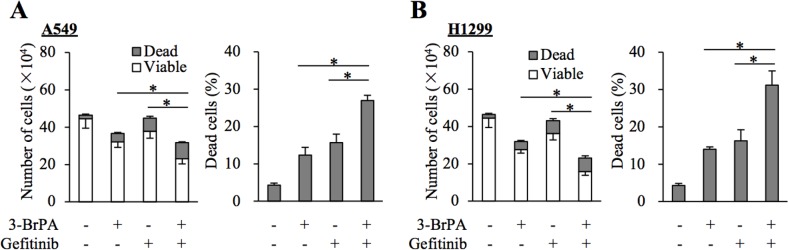
Pharmacological inhibition of hexokinase by 3-BrPA sensitizes resistant NSCLC cells to gefitinib **(A** and **B)** The indicated non-small-cell lung cancer (NSCLC) cells (1 × 10^5^) were treated with or without 10 μM gefitinib in the presence or absence of 10 μM 3-bromopyruvate (3-BrPA) for 3 days and then subjected to cell viability assay to determine the numbers of viable and dead cells (left panels) as well as the percentage of dead cells (right panels). Values in the graphs represent means and SD from three independent experiments. ^*^P < 0.05 [note that the numbers of viable cells are compared in the left panels of A and B].

We next asked whether GLUT1 contributes to the maintenance of gefitinib resistance through promotion of the subsequent glycolytic metabolism or through an as yet unknown function. To this end, we examined the effect of pharmacological inhibition of hexokinase, which catalyzes the initial step of glycolysis following intracellular transport of glucose [18]. The results indicated that the growth inhibitory effects of gefitinib on A549 and H1299 cells were augmented in the presence of 3-bromopyruvate (3-BrPA), a pyruvate analog targeting hexokinase 2 (Figure 4A and 4B), which was in support of the idea that active glucose metabolism driven by GLUT1-mediated glucose transport contributes to the gefitinib resistance of these cells.

### Glucose metabolism also contributes to the gefitinib resistance of cancer stem-like cells derived from EGFR-wt NSCLC cells and to erlotinib resistance

**Figure 5:**
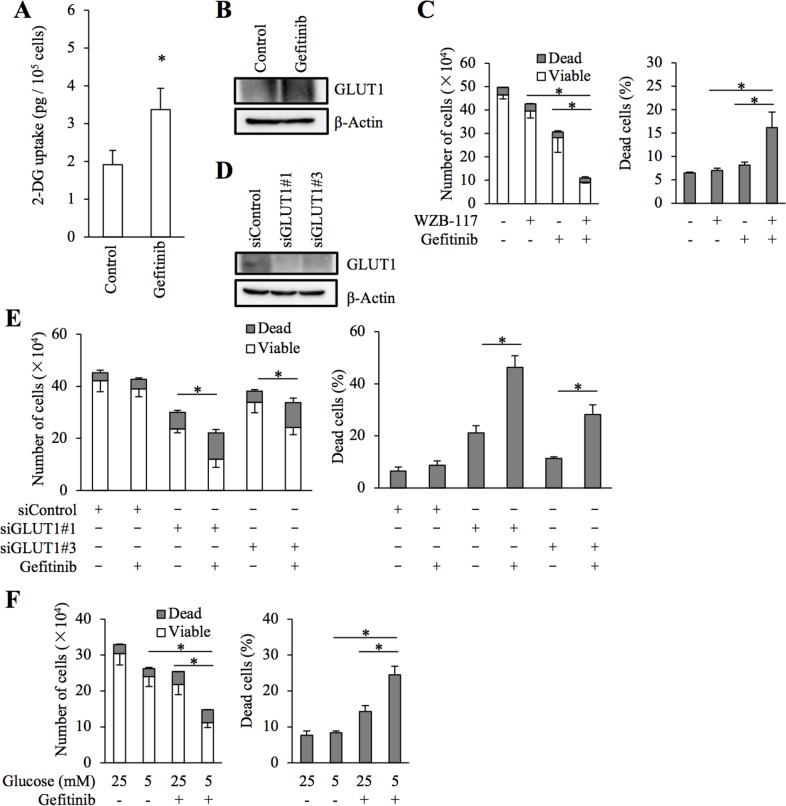
Role of facilitative glucose transport in gefitinib resistance of stem-like A549 cells **(A** and **B)** A549 CSLC cells cultured in the absence (Control) and presence of 10 μM gefitinib for 3 days were subjected to glucose uptake assay (A) and immunoblot analysis of GLUT1 expression (B). **(C)** A549 CSLC cells treated with or without 10 μM gefitinib in the presence or absence of 7.5 μM WZB-117 for 3 days were subjected to cell viability assay to determine the numbers of viable and dead cells (left panels) as well as the percentage of dead cells (right panels). **(D** and **E)** A549 CSLC cells were transfected with a non-targeting siRNA (siControl) or with either of the siRNAs against GLUT1 (siGLUT1#1 and siGLUT1#3) for 3 days. The cells were then subjected to immunoblot analysis of GLUT1 protein expression (D), or alternatively, treated with 10 μM gefitinib for another 3 days and subjected to cell viability assay to determine the numbers of viable and dead cells (left panels) as well as the percentage of dead cells (right panels) (E). **(F)** A549 CSLC cells cultured in the presence of the indicated concentrations of glucose were treated with or without 10 μM gefitinib for 3 days. Then, the cells were subjected to cell viability assay to determine the numbers of viable and dead cells (left panels) as well as the percentage of dead cells (right panels). Values in the graphs (A, C, E, and F) represent means and SD from three independent experiments. ^*^P < 0.05 [note that the numbers of viable cells are compared in the left panels of C, E, and F].

**Figure 6:**
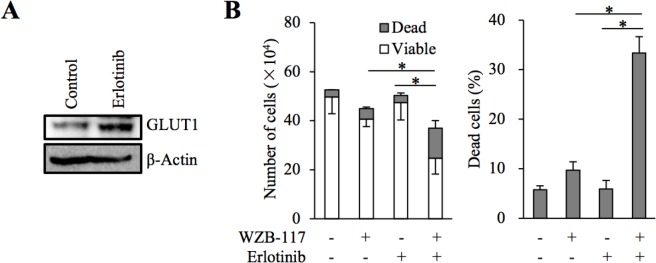
The role of GLUT1 in erlotinib resistance of A549 cells **(A)** A549 cells were treated in the absence (Control) and presence of 2 μM erlotinib for 3 days and subjected to immunoblot analysis of GLUT1 expression. **(B)** A549 cells (1 × 10^5^) were treated with or without 2 μM erlotinib in the presence or absence of 7.5 μM WZB-117 for 3 days and then subjected to cell viability assay to determine the numbers of viable and dead cells (left panel) as well as the percentage of dead cells (right panel). Values in the graphs represent means and SD from three independent experiments. ^*^P < 0.05 [note that the numbers of viable cells are compared in the left panel].

We have previously demonstrated that glucose metabolism mediated by GLUT1 is critically involved in the maintenance of stem cell properties of cancer stem-like cells [19]. We examined here whether GLUT1 is also involved in the control of gefitinib resistance in the cancer stem cell subpopulation of EGFR-wt NSCLC cells. To this end, we conducted essentially similar experiments with A549 CSLC, a cancer stem cell-like subline of A549 [20, 21], to those we did with parental A549 cells. The results showed that gefitinib increases glucose uptake accompanied by increased GLUT1 expression (Figure 5A and 5B) and that GLUT1 is critically involved in the gefitinib resistance of A549 CSLC cells (Figure 5C–5E). Intriguingly, lowering the glucose concentration in the culture medium mimicked the effect of GLUT1 inhibition in A549 CSLC cells (Figure 5F), further corroborating the idea that concentration-dependent, facilitative glucose transport has a key role in the maintenance of gefitinib resistance of these cells. We also examined whether the same mechanism is operative with erlotinib, another EGFR-TKI. Erlotinib sensitivity was significantly increased in the presence of WZB-117 (Figure 6), suggesting that the GLUT1-mediated resistance mechanism may not be unique to gefitinib.

### GLUT1 inhibition augments the anti-tumor effect of gefitinib *in vivo*

**Figure 7:**
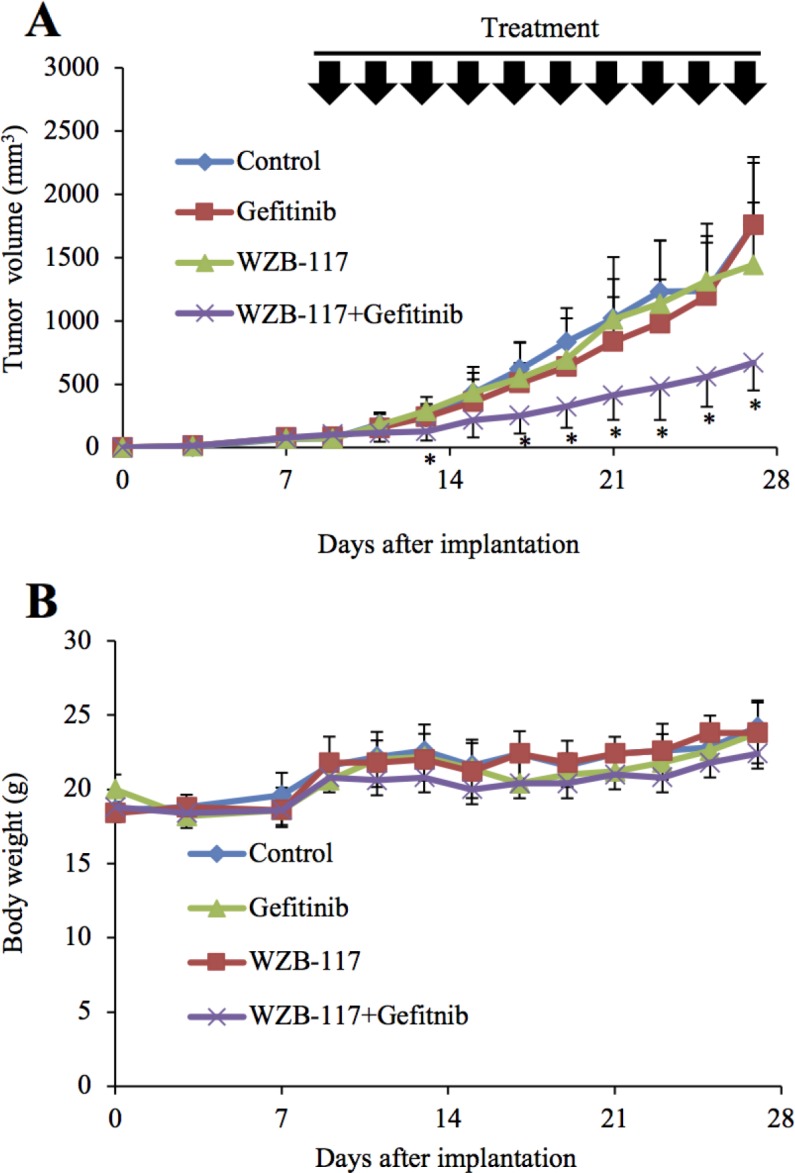
GLUT1 inhibition augments the anti-tumor effect of gefitinib *in vivo* Mice were implanted subcutaneously with A549 cells. After confirmation of tumor formation, treatment was initiated 9 days after implantation, and the mice were treated as indicated (with or without gefitinib [40 mg/kg, oral gavage] and WZB-117 [5 mg/kg, intraperitoneal injection]) at the indicated time points (every other day for a total of 10 times). Tumor volume **(A)** and mouse body weight **(B)** were measured, and the results are presented in the graphs as means and SD of each group (n = 10). Asterisks (^*^) are indicated at time points where the *P* values for comparisons between the WZB-117+Gefitinib group and the other three groups were all < 0.05.

The results obtained thus far suggested that GLUT1 inhibition effectively overcomes the gefitinib resistance of EGFR-wt NSCLC cells *in vitro*. To determine the therapeutic relevance of these *in vitro* findings *in vivo*, we tested the efficacy of systemic administration of gefitinib and WZB-117 alone or in combination against subcutaneous xenografts of EGFR-wt NSCLC cells. Since a pilot toxicity study showed that mice well tolerated the combination of oral gefitinib 40 mg/kg and intraperitoneal WZB-117 5 mg/kg given every other day for 20 days, we used this treatment protocol and treated tumors formed by subcutaneous implantation of A549 cells into nude mice. Strikingly, while neither gefitinib nor WZB-117 given alone had an appreciable effect compared to the control treatment, their combination significantly inhibited the growth of the tumors (Figure 7A) without affecting the general health status of the mice as assessed by their body weight (Figure 7B), suggesting that GLUT1 inhibition successfully sensitized otherwise resistant NSCLC cells to gefitinib *in vivo*.

## DISCUSSION

In this study, we have provided lines of evidence supporting the idea that GLUT1-mediated glucose metabolism is critically involved in gefitinib resistance of NSCLC. Our results indicated that both pharmacological and genetic inhibition of GLUT1 sensitized resistant NSCLC cells to the growth inhibitory effect of gefitinib. Inhibition of hexokinase, which catalyzes the first step of glucose metabolism following glucose uptake by cells, similarly sensitized resistant NSCLC cells to gefitinib. While our findings thus underscore the role of GLUT1-mediated glucose metabolism in gefitinib resistance, it may be worth noting here that, consistent with a previous observation that GLUT1 loss alone (i.e., without EGFR inhibition) was sufficient to inhibit the proliferation of NSCLC cells [12], GLUT1 inhibition per se, either pharmacological or genetic, had growth inhibitory effects, albeit modest, on NSCLC cells also in our study. Thus, the results suggest that GLUT1-mediated glucose metabolism may have two distinct but not mutually exclusive roles in NSCLC cells in that it not only contributes to gefitinib resistance but also to the growth of NSCLC cells as well.

Our demonstration that GLUT1 has pivotal roles in the growth and gefitinib resistance of NSCLC cells, combined with the observation that GLUT1 expression and glucose uptake were increased in resistant NSCLC cells after gefitinib treatment, may give rise to a hypothesis that the differential sensitivity of NSCLC cells to gefitinib could be attributed to the ability and inability of gefitinib to inhibit GLUT1 in each NSCLC cell. In support of this idea, the functional distribution of GLUT1 at the plasma membrane was shown to be lost upon erlotinib treatment in sensitive NSCLC cells but not in resistant cells [12]. Furthermore, it has been well documented *in vivo* through FDG-PET studies in mice and humans that early drop in glucose metabolism in NSCLC tumors after EGFR-TKI treatment is closely associated with their response to EGFR-TKIs, of which EGFR mutation status is a major determinant [22, 23]. Importantly, since GLUT1 has been shown to be critically involved in FDG uptake of NSCLC tumors [24], these previous findings suggest that activating EGFR mutations may promote EGFR-dependence of GLUT1 function in NSCLC. A hypothetical scenario could therefore be envisaged whereby EGFR inhibitors elicit in NSCLC cells a growth-inhibitory signal(s) subject to the GLUT1-dependent resistance mechanism, which is inactivated by EGFR inhibitors in NSCLC cells with activating mutations but not in those without, which then leads to the differential sensitivity of NSCLCs with and without activating EGFR mutations to EGFR inhibitors. However, since our preliminary data showed that glucose uptake of NSCLC cells without activating mutations may be inhibited by gefitinib at least partially for the first 24 h (unpublished data), it is conceivable that the duration and magnitude of glucose uptake inhibition in each individual cell, instead of the simple presence or absence of inhibition, may be a critical determinant of gefitinib sensitivity. As a limitation of our current experimental system of glucose uptake assay, we used mass-cultured cells and could analyze only viable cells. To test the hypothetical idea, therefore, future studies are expected to address at the single cell level the question of whether reduction of GLUT1 expression and glucose uptake actually precedes cell death in individual cells destined to undergo cell death, as well as the question of whether NSCLC cells with activating EGFR mutations are more susceptible to GLUT1 inhibition by gefitinib.

Although the detailed mechanisms by which GLUT1-mediated glucose metabolism confers resistance to EGFR inhibitors on NSCLC cells remain to be elucidated in this study, the above hypothetical scenario implies that GLUT1 inhibition may be an authentic approach to overcoming the primary resistance of NSCLC cells without activating EGFR mutations to EGFR inhibitors. Intriguingly, we also found in this study that GLUT1 was critically involved not only in primary gefitinib resistance associated with the lack of activating EGFR mutations but also in acquired resistance of NSCLC cells (i.e., PC-9-R cells), which were originally sensitive to gefitinib. These findings suggest that GLUT1-mediated glucose metabolism may be among the mechanisms behind acquired resistance and that its inhibition could be a promising strategy to overcome not only primary but also acquired resistance to EGFR inhibitors. Notably, our preliminary data indicate that GLUT1 inhibition sensitizes pancreatic and ovarian cancer cells to gefitinib (unpublished data). Thus, the GLUT1-dependent resistance mechanism may be operative across cancer types. Furthermore, recent reports have demonstrated that GLUT1 inhibition by use of WZB-117 sensitizes colon and breast cancer cells to conventional chemotherapeutic agents and radiation [25, 26, 27]. Collectively, these observations suggest that GLUT1-mediated glucose metabolism may possibly be involved in the control of a fundamental mechanism(s) of cellular survival and/or proliferation pertinent to different growth inhibitory stimuli in different cell types.

In the present study, the results of the mouse xenograft experiments demonstrated that systemic administration of a GLUT1 inhibitor WZB-117 in combination with gefitinib, neither of which had a discernible effect on the growth of tumors when administered alone, significantly inhibited the growth of NSCLC tumors. These *in vivo* results are quite in line with the *in vitro* findings and strongly suggest involvement of GLUT1-mediated glucose metabolism in gefitinib resistance of NSCLC not only *in vitro* but also *in vivo*. Importantly, the combination of WZB-117 and gefitinib did not appreciably affect the general health status of the mice as assessed by their body weight, indicating that the combination can exert an anti-tumor effect without causing toxicity. Thus, the results suggest that targeting GLUT1 may be a viable therapeutic approach to sensitizing otherwise resistant NSCLC tumors to gefitinib. Although the effect of erlotinib, another first-generation EGFR-TKI, on tumor growth *in vivo* in combination with the GLUT1 inhibitor is yet to be tested, our *in vitro* results suggest erlotinib would be effective similarly to gefitinib. Given the spreading use of second- and third-generation EGFR-TKIs such as afatinib and osimertinib in the management of NSCLCs with activating EGFR mutations [28, 29], it is also of interest whether GLUT1 inhibition sensitizes NSCLCs without such mutations to these new-generation EGFR-TKIs. Apparently, this should become a focus of future research. It also remains to be shown here whether GLUT1 is the optimal therapeutic target to control GLUT1-mediated glucose metabolism to sensitize NSCLC tumors with maximal safety and efficacy. In this respect, future elucidation of the mechanism by which GLUT1-mediated glucose metabolism contributes to EGFR inhibitor resistance is expected to lead to further identification of candidate therapeutic targets.

In conclusion, we demonstrated in this study that GLUT1-mediated glucose metabolism contributes to gefitinib resistance of NSCLC cells and that glucose uptake associated with GLUT1 expression remains active after gefitinib treatment in gefitinib-resistant NSCLC cells. We also demonstrated that GLUT1 inhibition effectively sensitizes EGFR-wt NSCLC tumors to gefitinib *in vivo*. Our findings, which suggest an intriguing possibility that failure of EGFR inhibitors to inhibit GLUT1-mediated glucose metabolism in EGFR-wt NSCLC cells might be a key mechanism behind their resistance to EGFR inhibitors, imply that concurrent inhibition of GLUT1 may be a rational and viable approach to overcoming the resistance of EGFR-wt NSCLC to EGFR inhibitors.

## MATERIALS AND METHODS

### Antibodies and reagents

Anti-β-actin (A1978) antibody and WZB-117 (SML0621) were purchased from Sigma (St. Louis, MO, USA). Anti-GLUT1 (07-1401) antibody was from Millipore (Billerica, MA, USA). Anti-GAPDH (#5174) antibody was purchased from Cell Signaling Technology, Inc. (Danvers, MA, USA). Gefitinib and erlotinib were purchased from Wako (Osaka, Japan). WZB-117, gefitinib, and erlotinib were dissolved in dimethylsulfoxide (DMSO) to prepare 10 mM, 10 mM, and 2 mM stock solutions, respectively.

### Cell culture and *in vitro* generation of a gefitinib-resistant cell line

Human NSCLC cell lines A549, H1299, PC-9, and HCC827 were purchased from the Riken BioResource Center (Tsukuba, Japan), and the authenticity of these cell lines was verified by subjecting cells in actual use to genotyping by short tandem repeat (STR) profiling (Bio-Synthesis Inc., Lewisville, TX, USA) followed by comparison with the American Type Culture Collection (ATCC) STR database for human cell lines. A549, H1299, and HCC827 were maintained in DMEM/F12 medium. PC-9 was maintained in RPMI1640. Normal human IMR90 fetal lung fibroblasts were purchased from ATCC and maintained in DMEM. The culture media were supplemented with 10% fetal bovine serum, 100 units/mL penicillin and 100 μg/mL streptomycin. All IMR90 experiments were performed using low passage number (< 8) cells. A549 CSLC cells, cancer stem-like cells derived from A549, were maintained under the monolayer stem cell culture condition as previously described [20]. Briefly, cells were cultured on collagen-I-coated dishes (IWAKI, Tokyo, Japan) in the stem cell culture medium (DMEM/F12 medium supplemented with 1% B27 [Gibco-BRL, Carlsbad, CA, USA], 20 ng/mL EGF and FGF2 [Peprotech Inc., Rocky Hill, NJ, USA], D-(+)-glucose [final concentration, 26.2 mM], L-glutamine [final concentration, 4.5 mM], 100 units/mL penicillin, and 100 μg/mL streptomycin). Stem cell culture medium was changed approximately every 3 days, and EGF and FGF2 were added to the culture medium every day.

A gefitinib-resistant subline of PC-9 (PC-9-R) was established by culturing PC-9 cells in escalating concentrations of gefitinib (1 μM - 5 μM) over a two-month period. PC-9-R cells were maintained in the presence of 5 μM gefitinib.

### Cell viability assay

Viable and dead cells were identified by their ability and inability to exclude vital dyes, respectively [19, 30]. In brief, harvested cells were stained with 0.2% trypan blue, and the numbers of viable and dead cells were determined using a hemocytometer. Cell viability (%) was defined as 100 × [number of viable cells/(number of viable cells + dead cells)], whereas the percentage of dead cells was defined as 100 × [number of dead cells/(number of viable cells + dead cells)].

### Gene silencing by siRNA

siRNAs against human GLUT1 (SLC2A1; #1:HSS109811, #3:HSS185757), Medium GC Duplex #2 of Stealth RNAi™ siRNA Negative Control Duplexes (as a negative control), and Lipofectamine RNAiMAX were purchased from Thermo Fisher Scientific (Waltham, MA, USA). Transfection of siRNAs was performed at a final RNA concentration of 100 nM using Lipofectamine RNAiMAX according to the manufacturer's instructions.

### Immunoblot analysis

Harvested cells were washed with ice-cold PBS and lysed in RIPA buffer (10 mM Tris-HCl [pH 7.4], 0.1% SDS, 0.1% sodium deoxycholate, 1% NP-40, 150 mM NaCl, 1 mM EDTA, 1.5 mM Na_3_VO_4_, 10 mM NaF, 10 mM sodium pyrophosphate, 10 mM sodium β-glycerophosphate and 1% protease inhibitor cocktail set III [Millipore]). After centrifugation for 10 min at 14,000 × g at 4°C, the supernatants were recovered as the cell lysates, and the protein concentration of the cell lysates were determined by the BCA protein assay kit (Pierce Biotechnology, Inc., Rockford, IL, USA). For immunoblot analysis of GLUT1 and its internal control, cells were lysed in lysis buffer (20 mM Tris-HCl [pH 7.4], 0.5% Triton X-100, 140 mM NaCl, 1 mM EDTA, 1.5 mM Na_3_VO_4_, 10 mM NaF, 10 mM sodium pyrophosphate, 10 mM sodium β-glycerophosphate and 1% protease inhibitor cocktail set III), followed by immediate addition of 1.5 (A549 and H1299) or 0.5 (PC-9-R) times the volume of Laemmli buffer 2x (125 mM Tris-HCl [pH 6.8], 4% SDS, and 20% glycerol) and subsequent sonication (UH-50, SMT CO., LTD, Tokyo, Japan) for 15 min at 4°C. After centrifugation for 10 min at 14,000 × g at 4°C, the supernatants were recovered as the cell lysates and boiled at 65°C for 20 minutes. Cell lysates containing equal amounts of protein were separated by SDS-PAGE and transferred to a polyvinylidene difluoride membrane. Membranes were probed with a primary antibody and then with an appropriate HRP-conjugated secondary antibody according to the protocol recommended by the manufacturer of each antibody. Immunoreactive bands were visualized using Immobilon Western Chemiluminescent HRP Substrate (Millipore). In principle, immunoblot analysis was repeated three times or more to ensure reproducibility.

### Glucose uptake assay

The glucose uptake assay was done essentially as described [31]. In short, cells were treated with 1 mM 2-deoxyglucose (2-DG) for 20 minutes. The reaction was stopped by harvesting the cells and washing them with ice-cold PBS 3 times. After removal of an aliquot for cell count, the cell pellet was solubilized in 10 mM Tris-HCl (pH7.4) by sonication (UH-50), followed by determination of the amount of 2-DG using 2DG Uptake Measurement Kit (OKP-PMG-K01, COSMO BIO Co., LTD, Tokyo, Japan), according to the manufacturer's instructions. Glucose uptake was calculated as the amount of 2-DG (pg) transported per 10^5^ cells in the 20 minutes in Figures 1A and 5A, and the relative values of glucose uptake compared with controls are shown in Figure 2G.

### Mouse study

Mouse xenograft studies were carried out essentially as previously described [19, 32]. For subcutaneous implantation, 5- to 8-week-old male BALB/ cAJcl-*nu*/*nu* mice (CLEA Japan, Inc., Tokyo, Japan) were, after being anesthetized with avertin (0.375 g/kg intraperitoneally), implanted subcutaneously in the flank region with A549 cells (5 × 10^6^) suspended in 200 μL PBS. After implantation, the recipient mice were monitored for general health status as well as for the presence of subcutaneous tumors. Tumor volume was determined by measuring tumor diameters (measurement of 2 perpendicular axes of tumors) using a caliper and calculated as 1/2 × (larger diameter) × (smaller diameter)^2^. For systemic administration of WZB-117, a stock solution of WZB-117 (1 mg/mL in DMSO) was diluted in PBS to prepare 200 μL solutions for each injection. The WZB-117 solutions were injected intraperitoneally to mice. Note that all the control- and WZB-117-treated mice received intraperitoneally an equal volume of DMSO per body weight (3.6 mL/kg/day). Similarly, for gefitinib administration, a stock solution of gefitinib (8 mg/mL in DMSO) was diluted in PBS to prepare 200 μL solutions for each injection, which were administered to mice by oral gavage. Note that all the control- and gefitinib-treated mice received orally an equal volume of DMSO per body weight (2.7 mL/kg/day). Drug treatment of mice was initiated after confirmation of subcutaneous tumor formation, and tumor-bearing mice were randomized into four groups before the initiation of drug treatment. All animal experiments were performed under a protocol approved by the Animal Research Committee of Yamagata University.

### Statistical analysis

The results are expressed as the mean and standard deviation (SD), and differences were compared using the 2-tailed Student's *t*-test, with Bonferroni correction for multiple comparisons. *P*-values < 0.05 were considered statistically significant and are indicated with asterisks in the figures.
